# Digital assessments for children and adolescents with ADHD: a scoping review

**DOI:** 10.3389/fdgth.2024.1440701

**Published:** 2024-10-08

**Authors:** Franceli L. Cibrian, Elissa M. Monteiro, Kimberley D. Lakes

**Affiliations:** ^1^ Fowler School of Engineering, Chapman University, Orange, CA, United States; ^2^ School of Education, University of California, Riverside, CA, United States; ^3^ Department of Psychology, College of Sciences, San Diego State University, San Diego, CA, United States; ^4^ Department of Psychiatry and Neuroscience, University of California, Riverside, CA, United States

**Keywords:** ADHD, assessment, digital health, computer, technology, attention, behavior, hyperactivity

## Abstract

**Introduction:**

In spite of rapid advances in evidence-based treatments for attention deficit hyperactivity disorder (ADHD), community access to rigorous gold-standard diagnostic assessments has lagged far behind due to barriers such as the costs and limited availability of comprehensive diagnostic evaluations. Digital assessment of attention and behavior has the potential to lead to scalable approaches that could be used to screen large numbers of children and/or increase access to high-quality, scalable diagnostic evaluations, especially if designed using user-centered participatory and ability-based frameworks. Current research on assessment has begun to take a user-centered approach by actively involving participants to ensure the development of assessments that meet the needs of users (e.g., clinicians, teachers, patients).

**Methods:**

The objective of this mapping review was to identify and categorize digital mental health assessments designed to aid in the initial diagnosis of ADHD as well as ongoing monitoring of symptoms following diagnosis.

**Results:**

Results suggested that the assessment tools currently described in the literature target both cognition and motor behaviors. These assessments were conducted using a variety of technological platforms, including telemedicine, wearables/sensors, the web, virtual reality, serious games, robots, and computer applications/software.

**Discussion:**

Although it is evident that there is growing interest in the design of digital assessment tools, research involving tools with the potential for widespread deployment is still in the early stages of development. As these and other tools are developed and evaluated, it is critical that researchers engage patients and key stakeholders early in the design process.

## 1 Introduction

Attention Deficit Hyperactivity Disorder (ADHD) is the most widespread psychiatric condition among children, affecting approximately 11.4% of children aged 3–17 years old in the United States (1). The societal costs associated with ADHD were estimated at $19.4 billion among children ($6,799 per child) and $13.8 billion among adolescents ($8,349 per adolescent) in the United States (2).

### 1.1 Gold standard assessments for a diagnosis of ADHD

A gold-standard diagnostic assessment of ADHD involves a comprehensive evaluation of symptoms related to inattention, hyperactivity, and impulsiveness (3). Inattention includes difficulty with focusing and maintaining attention, poor organizational skills, and forgetfulness. Behaviors often considered reflective of hyperactivity include: (1) movement behaviors (e.g., fidgeting, leaving seats when staying seated is expected, constant motion, restlessness) and (2) communication behaviors (e.g., talking nonstop, blurting out answers, interrupting others). Although gold-standard evaluations typically involve data from multiple sources (children, clinicians, parents, teachers) and multiple methods (standardized rating scales, structured and semi-structured clinical interviews, neuropsychological tests), in most parts of the world, these types of evaluations can often be difficult to obtain, are costly, and are not widely available.

In clinical practice, a diagnosis of ADHD is provided after a series of behavioral observations, combined with neuropsychological assessments and the completion of behavior rating scales by the individuals’ parent, guardian, or another informant. Self-reports of internal feelings and challenges experienced by the patient are also collected. Those reports can be influenced by factors intrinsic to the children themselves or extrinsic roles such as parents, the medical system, or school (4).

Unfortunately, scores derived using self-report, parent-, or teacher- report rating scales can be influenced by several factors (4), including rater bias, differences in behaviors across settings, and the relationship between the rater and the child (5). The limitations of rating scales have led to concerns about the validity of diagnoses, such as the potential for over-diagnosis, while barriers to gold-standard evaluations have raised concerns about under-recognition of ADHD. Failure to recognize and treat ADHD early on may adversely affect academic achievement (6), family and social relationships (7), employment (8), and functioning in other domains (4). Hence, there is a need to both increase the rigor and availability of diagnostic tools as well as the tools that could be used to assess progress in response to a variety of interventions. Given these challenges with assessment of ADHD symptoms, there is growing interest in increasing the rigor of diagnostic procedures as well as the assessment of progress in response to interventions using digital tools.

### 1.2 Towards a user-centered approach to develop assessment digital tools for ADHD

Currently, standardized assessment tools could go far to bolster the accuracy of diagnosis and the acceptance by families and others of the clinical diagnosis procedure, in which technology offers an opportunity to support human professionals and experts in their diagnostic and assessment work. Rapid technological advances in the last few decades have introduced tremendous opportunities to support professionals and experts in their diagnostic and assessment work. Despite these advances, only a handful of technology-supported assessment tools are used widely in practice. For example, the Continuous Performance Test (9) is one of the few computerized tests of attention that clinicians consistently use in their assessment battery during a neuropsychological evaluation.

On the other hand, research on digital tools has explored three main approaches to support the assessment and diagnosis of individuals with ADHD (10): (1) classify data from brain activity, either EEG or fMRI [e.g., (11–20)], (2) classify data collected from sensors (on the body, in the environment, or inherent to computational tool use) used during everyday activities and then create computational models that can classify unseen data instances [e.g., (21–27)], and (3) design and employ serious games or environments where users can play and interact (the interactions of the users with the game are analyzed to infer if the user has ADHD or related symptoms) [e.g., (28, 29)]. While the first approach considers only the data from brain activity of ADHD individuals without their input; the second and third approaches involve end-users to some degree in certain stages of the development process for a given digital assessment tool.

Recently, there has been a tendency to use user-centered, participatory, and ability-based design (30–35) and similar types of frameworks to include the needs and consideration of the primary end users through the whole process of designing, developing and evaluating digital tools to assess symptoms and behaviors, including ADHD (36–38). In the case of ADHD diagnosis and assessment tools, there are two primary end users that should be considered: the clinicians (psychologist, psychiatrist, among others) who are conducting the assessment, and the individuals (patients) who are performing the activities requested by the clinicians. Therefore, research needs to find ways in which people with ADHD and experts might be empowered through technology and included in research teams to develop assessment tools.

Given the early stages of research in this area, our goal in this research was to conduct a mapping review of digital assessments with the potential to diagnose and measure ADHD symptoms. A mapping review has been defined as a “preliminary assessment of the potential size and scope of available research literature” that “aims to identify the nature and extent of research evidence,” including ongoing research (39). Scoping reviews typically do not include a formal quality assessment and typically provide tables of findings along with some narrative commentary. They are systematic and can provide preliminary evidence that indicates whether a full systematic review (with quality assessment) is warranted at a given time.

## 2 Methods

Due to the breadth of the topic and our aims, we utilized the mapping review approach described by the Evidence for Policy and Practice Information and Coordinating Centre (EPPI-Centre), Institute of Education, London (40) and summarized by Grant & Booth (39). This method of review aims to map and categorize published scientific journal articles and reports to provide an overview of a particular field that can aid in identifying gaps in the evidence and directions for future research. Mapping reviews typically do not include meta-analysis or formal systematic appraisal but may characterize the strength of the evidence-based on the study design or characteristics (39). Grant & Booth (39) noted that mapping reviews are particularly helpful in providing a systematic map that can help reviewers identify more narrowly focused review questions for future work and potential subsets of studies for future systematic reviews and meta-analyses.

### 2.1 Data sources and searches

Following the PRISMA process for systematic reviews (41), we searched PubMed, Web of Science, ACM Digital Library, and IEEE Xplore for articles published in English from January 1, 2004, to January 1, 2024. With an interdisciplinary approach, we conducted this search using both the world's largest medical research database (PubMed), a multidisciplinary database (Web of Science), and the two largest databases for computed sciences (ACM Digital Library and IEEE Xplore). The Association for Computing Machinery (ACM) is the largest educational and scientific computing society in the world. The IEEE, an acronym for the Institute of Electrical and Electronics Engineers, has grown beyond electrical engineering and is now the “world's largest technical professional organization dedicated to advancing technology for the benefit of humanity.” (42). Together, the ACM and IEEE digital libraries comprise the vast majority of computing and digital indexing of publications from the organizations’ journals and conferences. We also reviewed the references from the included papers to identify additional relevant studies.

Our search strategy and search items are summarized in Table 1. We limited results to peer-reviewed research papers, excluding abstracts and short papers. Manuscripts were organized and reviewed using Zotero (an open-source reference management software). Keywords from retrieved articles are shown in Table 1.

**Table 1:** Systematic research strategy.

Domain	Search terms or database search limits
Population	ADHD OR Attention Deficit Hyperactivity Disorder
Topic	Assessment OR Diagnosis
Digital health interventions (Only for PubMed)	“digital” OR “computer assisted” OR “sensor” OR “mobile” OR “wearable” OR “smartphone” OR “tablet” OR “robot” OR “virtual reality” OR “augmented reality” “internet” OR “assistive technology” OR “computer intervention” OR “serious game” OR “web”
Search limits	Title, abstract, keywords, meta-data, years 2004–2024, no short papers, peer-reviewed, English only

### 2.2 Study selection

Study selection criteria are summarized in Table 2. We included research articles focusing on digital assessment for children and adolescents and excluded research focused on digital health interventions only. We included assessments for participants of all ages. We included assessments aimed for use by clinical settings, researchers, and community settings (e.g., schools). Importantly, we included assessments across various stages of development. To only focus on papers with empirical evidence regarding the use, adoption, usefulness, and effectiveness of digital assessment grounded by empirical evidence, we excluded papers focused solely on the theoretical design of technological tools, if they included no prototype or testing in individuals with ADHD.

**Table 2:** Mapping review study inclusion criteria.

	Included studies	Excluded studies
Population	• Individuals with ADHD (children, adolescents, adults)	• Studies that did not include individuals with ADHD
Study design	◦ Any experimental or quasi-experimental evaluative design, including pilot and feasibility studies ◦ Non-randomized studies (e.g., pre-post study with no control group) ◦ Cross-sectional studies, non-experimental studies ◦ Process evaluations without effect evaluation findings ◦ Case series or case studies	◦ Theoretical design or frameworks ◦ Studies not including individuals with ADHD or related participants (such as studies discussing a theoretical product or prototype with preliminary testing in a non-clinical group only).
Outcomes	• Focus on assessment in the following ADHD domains: ◦ Cognition/Attention ◦ Behavior Management/Self-Regulation ◦ Academic/Organizational Skills ◦ Motor Behaviors/physical activity ◦ Social/Emotional Skills ◦ Medication adherence ◦ Life/Vocational Skills	◦ Studies with a focus on intervention ◦ Studies that did not evaluate the use of an assessment or diagnostic tool ◦ Studies that focused on the use of machine learning to improve questionnaires ◦ Studies that focused on the use of machine learning to refine understanding of EEG or fMRI data
Publication type	• Peer-reviewed journal article, full paper proceedings, report.	• Conference abstract, study protocol, book, website, review, thesis or dissertation, short conference paper proceedings, posters, demos
Publication year	• From 1 January 2004 to 1 January 2024	• Before 2004, after 1 January 2024
Setting	• Any country or region.	
Language	• English	• Any other language

Two researchers reviewed abstracts and full papers and selected papers that both agreed met inclusion criteria. This process was completed two times to ensure accuracy. Further, inter-rater agreement was calculated on the basis of researchers’ categorization of articles using the previously mentioned inclusion and exclusion criteria (see Table 1). Researchers randomly selected 20% of the papers at the abstract review stage and coded the abstracts according to inclusion criteria. The agreement among researchers’ decision to include the article for these 20% of randomly selected articles was calculated to be greater than 80% (0.8125).

### 2.3 Evaluation of the stage of development of the assessment

This literature review includes research from diverse fields mainly including a clinical approach and a Computer Science/Human-Computer Interaction (HCI) approach. Therefore, literature on these fields follows different research lifecycles when developing technology in general and for assessment in particular (43). Typically, HCI researchers explore how emerging and commercially available technology can be designed and developed to support digital assessments using a user-centered approach and then conduct pilot feasibility studies to prove that the technology can be used in this context. On the other hand, clinicians validate digital assessments that follow a well-known or evidence-based theory (43) when conducting pilot testing and randomized control trials to validate the assessment. Thus, in this work, we proposed two categories to classify the digital assessment depending on the stage of development: (1) validated or (2) exploratory. The types of assessments that tended to fall in the ‘validated’ category for this sample of articles were computerized assessments commonly used by clinicians (e.g., Continuous Performance Test) that were adapted to develop a digital version. Alternately, the type of assessments that tended to fall in the ‘exploratory’ category were pilot studies, studies with few participants, and studies that were at the early stages of data collection, because these types of studies tend to involve the patients in early stages of the design of the tools as well as clinicians.

## 3 Results

**Figure 1:**
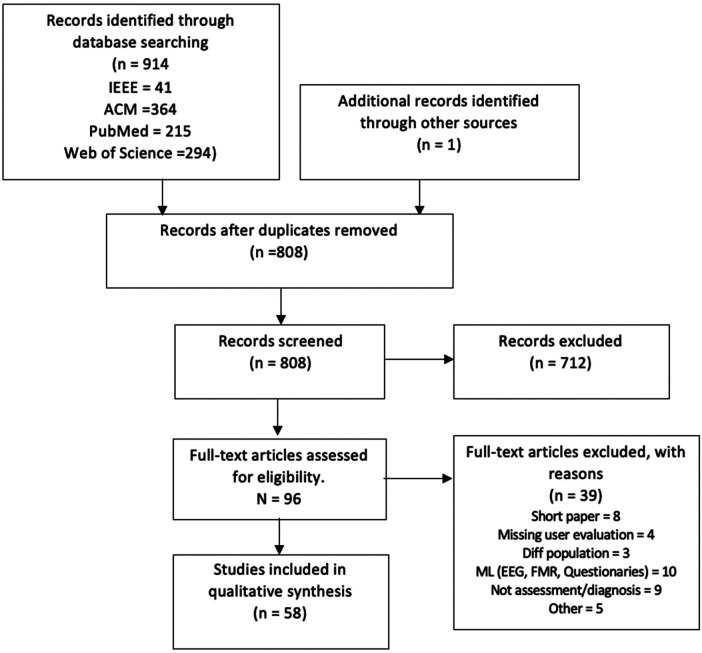
PRISMA flow diagram.

After applying the PRISMA (41) process for identifying appropriate articles for inclusion, our results are summarized in Figure 1. After duplicates were removed using Zotero, there were 808 records. Among those 808 records screened, 712 articles were excluded using the previously provided exclusion criteria. Then, 96 full-text articles were assessed for eligibility. Of those 96 full-text articles, 39 papers were excluded because they did not target assessment or diagnosis of ADHD, they did not present an evaluation of ADHD users, or they were *focused on the use of machine learning to improve questionnaires* [*i.e., utilizing machine learning to eliminate non-significant variables from psychometric questionnaires for ADHD diagnosis, aiming to reduce the administration time of these assessments* (44, 45)]. Ultimately, 57 papers were included in this mapping review (see Figure 1).

### 3.1 Targeted domains of cognitive and behavioral functioning

**Figure 2:**
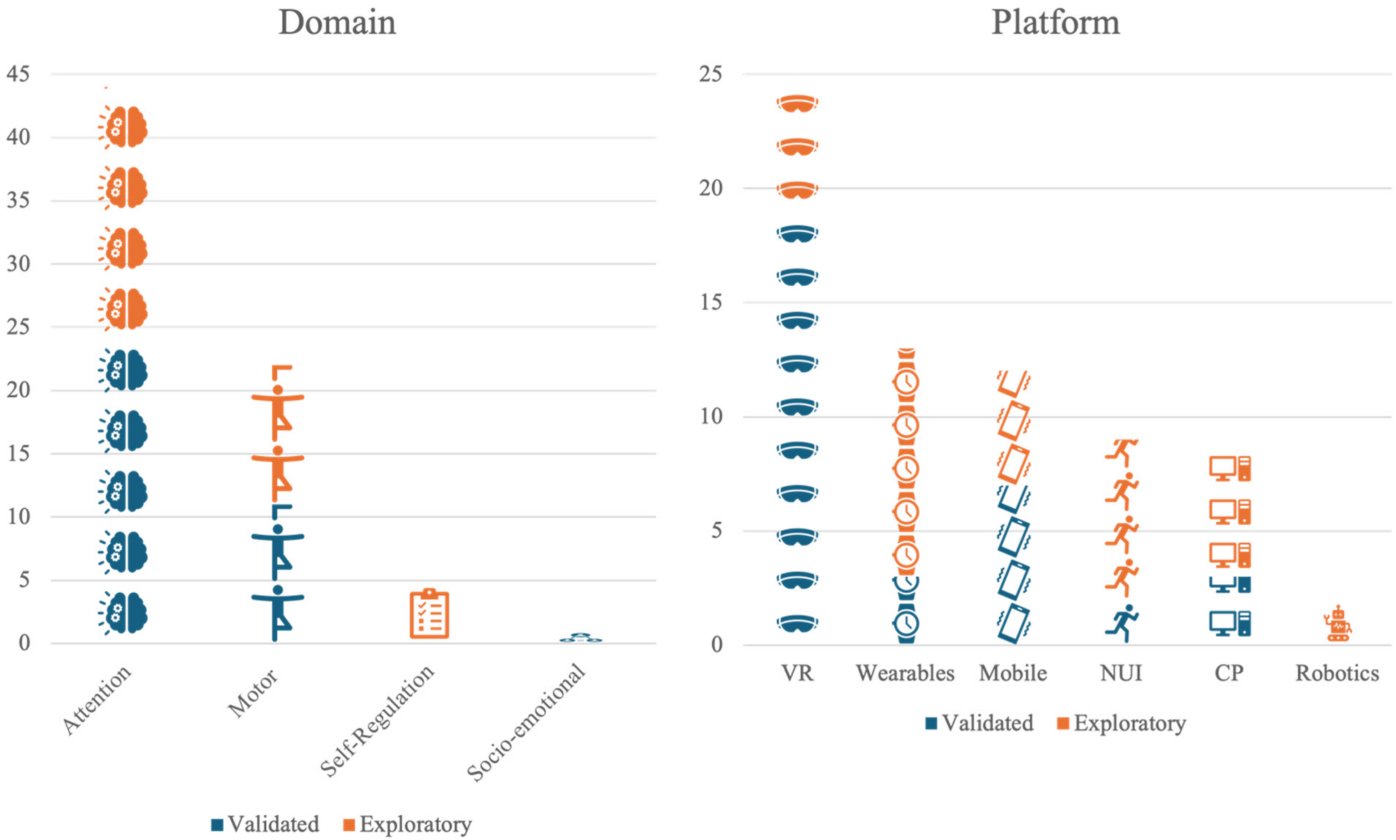
A pictograph showing the distribution of paper by domain (left) and by platforms (right). VR, virtual reality; NUI, natural user interfaces; CP, personal computer and web.

The domains of cognitive and behavioral functioning assessed by the tools in the included studies were grouped into four categories: cognition/attention, social/emotional skills, behavior management/self-regulation, or motor behaviors/physical activity (Figure 2-left).

Most papers (86%) described tools designed to assess attention and other aspects of cognition. Two main projects have been widely explored, the Virtual Reality Classroom, then called ClinicalVR (46–50), and AULA (51–53), which demonstrate the potential of VR in assessing attention. Even though VR has become more accessible, less expensive, less heavy, and more tolerable (e.g., it creates less motion sickness), it is still not particularly intuitive for many people. It may be totally out of reach for people with sensory challenges, including children with neurodevelopmental disorders like ADHD, but it provides a controlled environment to conduct assessments.

The second most common domain was motor behaviors or physical activity, with 43% of papers describing tools to assess behaviors in this domain. One approach is to use an indirect sensing device, such as depth cameras (e.g., Kinect), to track the movements. For example, Sempere-Tortosa and colleagues (2020) developed ADHD Movements, a computer software that uses the Microsoft Kinect V.2 device to capture 17 joints and evaluate the movements. A study with 6 subjects in a teaching/learning situation showed that there were significant differences in the movements between the ADHD and control group (54).

On the other hand, Muñoz-Organero and colleagues (2018) tested direct sensing (e.g., wearables) using accelerometers on the dominant wrist and non-dominant ankle of 22 children (11 with ADHD, 6 of whom were also medicated) during school hours. They used deep learning algorithms [e.g., Convolutional Neural Network (CNN)] to recognize the movement differences between nonmedicated ADHD children and their paired controls. There were statistically significant differences in the way children with ADHD and those without moved for the wrist accelerometer, but only between nonmedicated children with ADHD and children without ADHD for the ankle accelerometer.

None of the papers described tools designed to measure social/emotional functioning, and only one paper described a tool designed to measure behavior management or self-regulation (28). Their research group developed a Contextualized and Objective System (COSA) to support ADHD diagnosis by measuring symptoms of inattention, hyperactivity, and impulsivity. Impulsivity is often used as a proxy for measuring self-regulation using performance-based tasks, or Serious Games. The Serious Games developed for COSA were informed by existing auxiliary diagnostic performance-based tasks of inhibition and impulsivity, including CPT, Go/No-Go, and the Matching Familiar Figures Test. The COSA instruments were used to measure inhibition and impulsivity (e.g., stop yourself from eating “eCandy”).

A hybrid approach, meaning the measurement of multiple symptoms, using multiple digital tools (e.g., wearable sensor, intelligent hardware, paired with mobile application), has also been explored to create a system to support the assessment of multiple domains of ADHD (e.g., attention and hyperactivity). An initial pilot study to investigate children's attentional control in a VR classroom was combined with instruments to detect “head turning” and gross motor movements. These instruments included motoric tracking devices on the VR head-mounted display and wearable hand and leg tracking systems (47). Combining the use of VR, Serious Games, and motor behaviors allowed Parsons and colleagues (2007) to predict the body movements a hyperactive child may be engaging in the classroom. Similarly, the WEDA system, tested with 160 children ages 7 to 12, half with ADHD, attempted to discriminate between challenges in inattention from those related to hyperactivity and impulsivity, finding that the tasks cover all symptoms but perform better related to inattention (29). Overall, the summarized works suggest that it is possible to assess several ADHD-related behaviors using a multimodal technology approach. However, it is unclear how we can refine the current assessment tools to collect data augmented with contextual, or real-life, information.

### 3.2 Technology platforms applied to ADHD assessment

Among the included studies, the technology platforms used in the assessment process were varied: virtual or augmented reality (49%), natural user interfaces (17%), personal computers (17%), mobile devices or tablets (23%), sensor/wearables/EEG (25%), and robotics (3%) (Figure 2-right). *Virtual and augmented reality* is a rapidly shifting label in the literature, but for the purpose of this review, studies assigned to that category included fully immersive virtual reality as well as mixed-reality and augmented reality approaches. This category included virtual worlds and immersive video games. *Natural user interfaces* included the use of input devices beyond traditional mice and keyboards, such as pens, gestures, speech, eye tracking, and multi-touch interaction. The *personal computers* category included applications that require a traditional keyboard, mouse/touchpad, and monitor. *Mobile devices and tablets* can access such applications, as well, but this category was reserved for so-called “mobile first” applications, focused on an intentional design towards mobility. *Sensors and wearables* include the use of automated sensing technologies, such as accelerometers, heart rate sensors, microphones, and brain-computer interfaces, both in the environment and on the body. *Robotics*, a similarly broad and dynamic category, included physical instantiations of digital interactions, such as both humanoid or anthropomorphic robots and general digital devices that carry out physical tasks. This grouping included autonomous robots and those operated remotely by humans.

### 3.3 Stage of development

Results from the literature review revealed that 27 of the projects that met inclusion criteria were considered “validated assessments”. The projects with validated assessment studies tended to adopt widely accepted assessments, such as the Continuous Performance Test (55), and implemented the assessment within a novel digital environment, such as via virtual reality (see Table 3). For example (51, 58, 75), all administered the CPT within a virtual reality classroom environment. In some cases, these widely used assessments seemed to inspire ideas for the measurement of symptoms of ADHD (e.g., attentional control, inhibition, reaction time) in a gamified virtual environment (e.g., the Nesplora Aquarium test) (52). Other studies in this stage of development administered widely used assessments via telemedicine. For example, Sabb and colleagues (2013) administered the Stroop task (76) via a web-based platform typically used to meet patients virtually.

**Table 3:** Clinically validated assessments included in this review.

Study	Digital health assessment							Domain				User		Setting				Technology platform					
	Telemedicine	Wearables/sensors	Web-based assessment	Virtual reality	Serious games	Robot assistant	Computer application/sensing in the environment	Cognition/attention	Social/emotional skills	Behavior management/self-regulation	Motor behaviors/physical activity	Caregivers (parents/teacher)	Clinician/researchers	Home	School	Lab	Clinic	Personal computers & web	Mobile devices or tablets	Sensors/wearables/EEG	Virtual & augmented reality	Robotics	Natural user interface
Adamou et al. (56)	X							X					X				X	X	X				
Adams et al. (57)				X				X			X		X			X					X		
Areces et al. (51)				X				X					X			X				X	X		
Areces et al. (58)				X				X					X			X				X	X		
Camacho-Conde and Climent (52)				X				X			X		X			X			X		X		
Coleman et al. (46)				X				X					X			X					X		
Díaz-Orueta et al. (53)				X				X			X		X			X					X		
Eom et al. (59)				X				X					X			X					X		
Gutierrez-Maldonado et al. (60)				X				X					X			X					X		
Hyun et al. (61)							X	X					X			X			X				
Iriarte et al. (62)				X				X			X	X	X			X					X		
Johnson et al. (63)							X				X		X			X			X				X
Lalonde et al. (64)				X				X					X			X					X		
Leitner et al. (65)	X							X			X		X			X		X					
Loskutova et al. (66), Loskutova et al. (67)			X					X	X				X				X		X				
Mangalmurti et al. (68)				X				X					X			X					X		
Muhlberger et al. (69)				X				X			X		X			X					X		
Mwamba et al. (70)					X						X		X			X			X				X
Neguț et al. (71)				X				X					X			X					X		
Nolin et al. (72)				X				X			X		X		X						X		
Parsons et al. (47)				X				X					X			X					X		
Pollak et al. (48)				X				X					X			X					X		
Rodriguez et al. (51)				X				X					X			X				X	X		
Sabb et al. (73)	X		X					X					X	X				X					
Zulueta et al. (74)				X				X			X		X			X					X		

On the other hand, 26 of the projects were considered “exploratory assessments” as they were either pilot studies, studies with small sample sizes, or the research team was in the early stages of data collection, and the primary goal was to launch the assessment tool rather than collect usable data. The studies aimed at refining exploratory assessments using novel technological platforms or a combination of the following platforms, including (a) personal computers and the internet (24%), (b) mobile devices or tablets (20%), (c) sensors, wearables, or EEG (40%), virtual reality (24%), robotics (8%), and a natural user interface (28%) (see Table 4). These types of technologies were created by researchers to better meet the needs of participants (patients and clinicians), as current commercial devices may not provide the sensors and feedback needed to conduct in-depth assessments of ADHD symptoms in accordance with current American Psychological Association diagnostic criteria [DSM−5; (3)]. However, the approach of exploratory research is first to develop the technology and provide evidence that it is feasible for application, once feasibility is established, the primary objective of researchers is to test the usability of the assessment data collected for either patients or clinicians. For example, Son and colleagues (2021) are in the early stages of developing an ‘objective diagnosis of ADHD by analyzing a quantified representation of the action of potential patients in multiple natural environments’. The research team applies the diagnostic criteria for ADHD listed in the DSM-5 (3) to virtual reality and artificial intelligence applications in order to build an AI model which will classify the potential patient as having either ADHD inattentive type, hyperactive-impulsive type, combined type, or no diagnosis. Future steps for this research may include comparing the decisions of the AI model to those of a clinician in a clinical trial.

Overall, it is ideal to combine both approaches. To better conceptualize this goal, it is useful to position these approaches on a continuum of digital health technological tool development where stages of development from exploratory (early stages often piloted by human-computer interaction researchers) to validated (late stages such as clinical trials led by clinicians) lie. Thus, researchers in the field should strive to recruit multidisciplinary teams that are capable of implementing methodologies that combine both approaches over the course of a tool's developmental lifetime. When made, these proposed changes will accelerate the validation and widespread use of diagnostic digital health technologies for ADHD.

**Table 4:** Exploratory assessments included in this review.

Study	Digital health assessment							Domain				User		Setting				Technology PLATFORM					
	Telemedicine	Wearables/sensors	Web-based assessment	Virtual reality	Serious games	Robot assistant	Computer application/software/sensing in the environment	Cognition/attention	Social/emotional skills	Behavior management/self-regulation	Motor behaviors/physical activity	Caregivers (parents/teacher)	Clinician/researchers	Home	School	Lab	Clinic	Personal computers & web	Mobile devices or tablets	Sensors/wearables/EEG	Virtual & augmented reality	Robotics	Natural user interface
Aflalo et al. (77)							X	X					X			X			X				
Arakawa et al. (78)		X								X	X	X	X	X					X	X			
Brkic et al. (79)					X			X				X	X			X			X				
Cazzato et al. (80)							X	X								X							X
Chen et al. (28)		X					X	X		X		X	X			X		X		X			X
Cho et al. (81)		X		X				X			X		X			X				X	X		
Delgado-Gómez et al. (82)					X			X					X				X						X
Gardner et al. (83)		X						X					X			X			X				
Jiang et al. (29), Luo et al., (84)		X			X		X	X		X	X		X			X		X		X			X
Kim et al. (85)		X						X		X			X			X				X			
Krichmar and Chou (86)						X					X				X							X	
Lee et al. (87), Lee et al. (88), Lindhiem et al. (89)		X				X		X			X	X				X				X		X	X
Loleska and Pop-Jordanova (90)		X						X			X	X	X			X		X	X				
Muñoz-Organero et al. (25)		X									X		X		X					X			
Santos et al. (91)					X		X	X							X	X		X					
Seesjärvi et al. (92), Jylkkä et al. (93), Merzon et al. (94)			X					X					X			X		X					
Sempere-Tortosa et al. (54)		X					X				X					X							X
Son et al. (95)				X				X					X			X					X		
Stokes et al. (96)		X		X				X					X			X				X	X		
Ulberstad et al. (97)	X		X					X			X		X	X		X		X					X
Wehmeier et al. (98)		X						X			X		X			X				X			
Wiguna et al. (100)				X				X		X	X		X		X	X					X		
Yeh et al. (50)				X				X					X			X					X		

## 4 Discussion

Recent estimates suggest that nearly 11% of children and adolescents in the United States experience ADHD symptoms (1). Thus, there is a significant need to broaden access to evidence-based treatments to support individuals with ADHD. In this paper, we argue that digital health assessments have the potential for widespread impact on the assessment infrastructure necessary to connect individuals with ADHD to the necessary treatments designed to support them. This scoping review addresses a critical gap in the literature and illustrates the growing international interest in digital health assessment for ADHD. Many of the excluded papers in our search described novel digital health assessment tools that were not sufficiently developed or have yet to be evaluated. This suggests that this field of research will continue to grow rapidly and, therefore, intentional investment in translation from early designs for digital assessment tools to robust products as well as from pilot studies to larger scale clinical trials are necessary next steps to meet the needs of the field.

### 4.1 Participant engagement and user-centered assessment

Involving the final users in the development of the assessment is a crucial step in trying to create unbiased digital tools. Therefore, different points of view should be held up to the light. Traditionally, clinicians are charged with developing the assessment tools for ADHD, and subsequently, researchers in the technological fields “translated” those tools into digital health assessments. The advantage of this approach is that the tools tend to be more widely “accepted” by other clinicians as they use “validated” assessments to evaluate symptoms and behaviors without input from the patients who are responsible for conducting the activities requested by the clinicians.

On the other hand, studies have reported conducting interviews with one (29) or more (78) clinicians to incorporate their perspectives before building the tools. Additionally, these studies consider or evaluate patient and caregiver satisfaction (99) prior to deploying the actual tool, showing an initial commitment to following a user-centric approach instead of translating current theories into digital interventions [e.g., (53, 62)]. Unfortunately, developing quality assessment tools is time-consuming and starts from co-design sessions before developing low and high-fidelity prototypes. The first evaluation of those prototypes targets the tools’ feasibility and usability before developing the final tools that can be then “validated.” While this approach is highly recommended, the approach neglects to answer important research questions that have yet to be answered, including how to engage ADHD participants in the co-design sessions, how to balance their needs with the clinicians’ needs, how the collected data can be used fairly, and what should need to be done to transform those prototypes into valid assessment.

### 4.2 Clinical implications

The underdiagnosis of ADHD results in patients not receiving treatment, which poses psychological, financial, academic, and social burden to the patient and their community (101). Further, failure to diagnose ADHD prevents children and their families from getting the assistance necessary to achieve their full potential in academic, workplace, and psychosocial settings (102). A lack of diagnosis can lead to a lack of treatment and restricted access to accommodations that will have a cascade of consequences for an individual's academic achievement (6), family and social relationships (7), employment (8), and other critical components of life success (4).

The clinical implications for the development of diagnostic digital health technologies to diagnose ADHD are vast and varied. To support access to digital assessment tools researchers will need to adopt rigorous approaches to ensure the development of reliable and feasible tools designed to be used by clinicians who seek to evaluate ADHD symptoms and diagnose ADHD. Innovative computational approaches paired with expert human decision-making have the potential to improve the quality of assessments while decreasing their costs. Thus, novel technologies can support clinicians through the collection of data from multiple modes of assessment that support the decision-making of the experts and improve the accuracy of diagnosis.

### 4.3 Future research directions

Only a handful of studies collected from this scoping review examined products that were designed using user-centered participatory and ability-based design methods. User-centered, participatory and ability-based design frameworks demand two parallel approaches: inclusion of the needs and consideration of the primary end users of these technologies early in the design process and consideration for the ways in which people with ADHD might be empowered through technology and included in research teams. In our own work, we strive to include children and adolescents with ADHD on our design teams, engaging them in creating their own inventions as well as commenting on and critiquing ours (103–108). Although these approaches are time consuming and can be more challenging to implement, the long-term adoption and ultimate success of digital health tools requires the input and perspectives of those who experience the conditions as well as other relevant stakeholders.

In terms of hyperactivity, research has shown that measuring and predicting movement-related behaviors using data gathered from wearables and cameras to assess ADHD is feasible and helps to understand more about the role of hyperactivity in motor performance. However, the assessment should also consider the core features of ADHD related to attention, socioemotional functioning, and self-regulation. Therefore, a multimodal approach should be adopted.

A possible reason for the lack of tools designed to measure social/emotional functioning and self-regulation is the challenge of eliciting the real-life emotions involved in behavioral management and self-regulation, especially with respect to the demands placed on children with neurodevelopmental disorders in schools and at home. Take for example a child who is being bullied by peers at school or a child who has difficulty reading and, therefore, cannot access the academic material and becomes frustrated in a classroom. These are challenges children with ADHD are faced with daily and it is possible Serious Games have not yet been developed to tap into these charged and challenging socioemotional and behavioral contexts.

### 4.4 Study limitations

There are several limitations to consider when reading this mapping review. First, we limited our review to papers published in English language journals and to PubMed, IEEE, and ACM databases. Although these databases contain the largest collections of research in the field and can be considered comprehensive for scholarly publications in English, limiting the search to articles published in English and to articles available through these databases has inherent limitations. For example, it excludes grey literature, which includes white papers that are not peer-reviewed but that can be common surrounding consumer products. We excluded papers that developed diagnostic assessments for multiple diagnostic groups other than ADHD (e.g., for individuals with Autism who also exhibited symptoms of ADHD, children who demonstrate self-regulation difficulties). We also excluded papers for which the focus was on digital health intervention and treatment, rather than assessment. Finally, the broad range of terms used in this space makes a true comprehensive review incredibly difficult. Keyword selection, terminology usage, and digital libraries in the mHealth space are not consistent within disciplines, across disciplines, nor across countries. Despite these limitations, our work provides a map of the current scientific work in this space that can aide clinical and computing scientists in identifying gaps and potential targets for future work.

## 5 Conclusion

Recently, there has been rapid growth in collaboration between the fields of computing and clinical sciences. Given the explosion in telehealth and telemedicine since the beginning of the COVID-19 pandemic, this growth underscores the need for empirically and well-developed technological diagnostic tools. This mapping review highlights current work on the development of diagnostic tools used to assess symptoms of ADHD, providing examples of how emerging technologies can enhance diagnostic processes for both researchers and clinicians.

The included studies show that while some diagnostic technologies seem promising, there are still opportunities that should be addressed to widespread clinical use. Specifically, future work should focus on:

1. User-Centered Design: Emphasizing user-centered design strategies to tailor diagnostic tools to the needs and experiences of clinicians, patients, and caregivers, thereby improving acceptability and usability.
2. Interdisciplinary Collaboration: Fostering multidisciplinary collaboration between computer science, HCI researchers, clinicians, and other stakeholders to bridge gaps in knowledge and practice, ensuring that technological advancements are clinically relevant and evidence-based.
3. Integration with Clinical Workflow: Developing strategies to seamlessly integrate diagnostic technologies into existing clinical workflows, ensuring they complement rather than disrupt standard practices.
4. Rigorous Validation: Conducting comprehensive validation studies to ensure the accuracy, reliability, and effectiveness of diagnostic technologies in diverse clinical settings.

## Data Availability

The original contributions presented in the study are included in the article/Supplementary Material, further inquiries can be directed to the corresponding author.
