# Corrigendum: Digital assessments for children and adolescents with ADHD: a scoping review

**DOI:** 10.3389/fdgth.2024.1528500

**Published:** 2024-11-28

**Authors:** Franceli L. Cibrian, Elissa M. Monteiro, Kimberley D. Lakes

**Affiliations:** ^1^ Fowler School of Engineering, Chapman University, Orange, CA, United States; ^2^ School of Education, University of California, Riverside, CA, United States; ^3^ Department of Psychology, College of Sciences, San Diego State University, San Diego, CA, United States; ^4^ Department of Psychiatry and Neuroscience, University of California, Riverside, CA, United States

**Keywords:** ADHD, assessment, digital health, computer, technology, attention, behavior, hyperactivity

A Corrigendum on Digital assessments for children and adolescents with ADHD: a scoping review By Cibrian FL, Monteiro EM, Lakes KD (2024). Front. Digit. Health. 6:1440701. doi: 10.3389/fdgth.2024.1440701

In the published article, an author name was incorrectly written as Kimbelery D. Lakes. The correct spelling is Kimberley D. Lakes.

The authors apologize for this error and state that this does not change the scientific conclusions of the article in any way. The original article has been updated.

